# GSK3β modulates NF-κB activation and RelB degradation through site-specific phosphorylation of BCL10

**DOI:** 10.1038/s41598-018-19822-z

**Published:** 2018-01-22

**Authors:** Ali Abd-Ellah, Cornelia Voogdt, Daniel Krappmann, Peter Möller, Ralf B. Marienfeld

**Affiliations:** 1 Institute of Pathology, University of Ulm, Albert-Einstein-Allee 23, 89070 Ulm, Germany; 2 Research Unit Cellular Signal Integration, Institute of Molecular Toxicology and Pharmacology, Helmholtz Zentrum München - German Research Center for Environmental Health, Neuherberg, Germany; 3 Department of Pathology, Qena Faculty of Medicine, South Valley University, Qena, 83523 Egypt

## Abstract

Glycogen synthase kinase 3β (GSK3β) is a ubiquitously expressed serine/threonine kinase involved in the regulation of various cellular functions, such as energy homoeostasis, cell growth and developmental processes. More recently, GSK3β has been identified as a part of a protein complex involved in the regulation of the CARMA1-BCL10-MALT1 complex (CBM complex) formation, which is a key signalling event upon antigen receptor engagement of B and T cells, required for the activation of the NF-κB and JNK pathways. However, conflicting reports have been published regarding the role of GSK3β for the activation of the NF-κB signalling pathways. Therefore, we aimed to determine the impact of GSK3β on the NF-κB signalling induced upon T cell activation. Blocking GSK3β by either pharmacologic inhibitors (SB216763 and SB415286) or by RNAi caused a reduced proteolysis of the MALT1 targets CYLD1, BCL10 and RelB as well as diminished IκBα degradation, NF-κB DNA binding and NF-κB activity. This negative effect on NF-κB appears to be due to a diminished CBM complex formation caused by a reduced BCL10 phosphorylation. Taken together, we provide here evidence for a novel regulatory mechanism by which GSK3β affects NF-κB signalling in activated T cells.

## Introduction

Engagement of the antigen receptors, T cell receptor (TCR) in case of T cells and B cell receptor (BCR) in case of B cells, induces the formation of a higher molecular weight complex, composed of the MALT1-BCL10 dimer and CARMA1, thus forming the CARMA1-BCL10-MALT1 complex (CBM complex). The CBM complex serves as a platform for the subsequent activation of several downstream signal transduction pathways, including the NF-κB and the JNK signalling pathways^1–3^. CBM complex formation is regulated by a variety of phosphorylation events primary occurring at CARMA1. Protein kinase C isoforms (PKCs) have been shown to be the most important CARMA1 kinases, although other kinases like HPK1, AKT1, or CK1alpha are also capable of CARMA1 phosphorylation^4–6^. Phosphorylation of BCL10 also contributes to the regulation of the CBM complex formation^7^. IKK2 has been shown to phosphorylate BCL10 at a set of serine residues (Ser^134^, Ser^136^, Ser^138^, Ser^141^, and Ser^144^) in the center of the protein. This IKK2 mediated BCL10 phosphorylation exerts a dual function: Firstly, it is required for the formation of the CBM complex and has thus a positive effect on NF-κB activation. Secondly, IKK2-mediated BCL10 phosphorylation weakens the BCL10-MALT1 interaction, which is crucial for the function of the CBM-complex. Thus, IKK2 mediated BCL10 phosphorylation appears to be a negative feedback mechanism limiting the signal duration. In essence, IKK2 mediated BCL10 phosphorylation exerts both a positive as well as a negative effect on the CBM complex formation and subsequent NF-κB activation.

MALT1 is required for activation of the canonical NF-κB pathway induced upon TCR or BCR engagement. As a scaffolding protein, MALT1 mediates IKK complex activation and NF-κB activation through recruitment of downstream effector proteins as ubiquitin ligase TRAF6^8^. A second mechanism that increase the duration and amplitude of NF-κB activation is through MALT1 proteolytic activity were MALT1 cleaves NF-κB inhibitory proteins RelB^9^ and A20^10^. The RelB proteolysis is a two-step process, initiated by an endoproteolytic cleavage at position Arg^85 ^^9,11^, removing an amino terminal leucine zipper, followed by the subsequent degradation of the remaining instable RelB protein (ΔRelB) via the proteasomal pathway. However, A20 and RelB are not the only targets of the MALT1 endoprotease activity. Another targets are BCL10, haem-oxidized IRP2 ubiquitin ligase 1 (HOIL-1), Regnase and Roquin 1, and Cylindromatosis (CYLD1), whose cleavage is required for c-Jun N-terminal kinase (JNK) pathway activation upon T cell activation^12–14^. Although the proteolytical steps leading to RelB degradation have been unravelled, it still remains not completely understood how the signal-induced RelB degradation is regulated. Phosphorylation of murine RelB at Thr^84^ and Ser^552^ coincides with its degradation and a RelB mutant carrying T84A and S552A substitutions appears to be more stable in activated T cells^9^. Phosphorylation of Ser^552^ (Ser^573^ in human RelB) can be catalysed by the protein kinase GSK3β. Moreover, GSK3β forms a complex with RelB even in resting T cells and blocking GSK3β by either a pharmacological inhibitor or by a siRNA mediated knock down impairs the signal-induced RelB degradation^15^. Of note, all these site-specific RelB phosphorylations affect the first step of RelB degradation while the second, proteasome-dependent step appears to occur automatically upon removal of the amino-terminus.

Interestingly, GSK3β was also found to be recruited together with other β-catenin destruction complex components to activated CARMA1^16^. However, which function this CBM complex recruited GSK3β exerts is not fully understood although previously published studies suggest an impact of GSK3β on NF-κB signalling. GSK3β deficient mice, for instance, showed embryonic death due to massive apoptosis in the liver, similar to IKK2 and RelA deficient mice^17–19^. Moreover, embryonic fibroblasts derived from GSK3β deficient mice showed apoptosis upon TNFα stimulation being unable to activate NF-κB^17^. In addition, another study showed that GSK3β affects NF-κB target gene expression in a gene specific manner by controlling promoter-specific recruitment of NF-κB^20^.

As previously published results emphasize the importance of CBM complex formation for RelB degradation^15^, we analysed the potential role of the RelB regulator GSK3β for CBM complex formation. As expected, RelB degradation in PMA + ionomycin (P/I) or anti-CD3/CD28 stimulated Jurkat T-ALL cells was diminished upon blockage of GSK3β. However, GSK3β inhibition also distinctively impaired the proteolysis of additional substrates of the MALT1 para-caspase activity, like CYLD1 or BCL10. Moreover, P/I induced NF-κB activation as monitored by IκBα degradation, NF-κB DNA binding or NF-κB luciferase reporter activity was also diminished after GSK3β blockage. This reduced NF-κB activity observed upon GSK3β inhibition appears to be due to a reduced CBM complex formation and BCL10 phosphorylation. Mechanistically, GSK3β appears to phosphorylate BCL10 similar to IKK2. In essence, we identify GSKβ as a novel regulator of the antigen receptor induced CBM-complex formation and canonical NF-κB activation.

## Results

### GSK3β is required for signal induced MALT1 endoprotease activity

**Figure 1:**
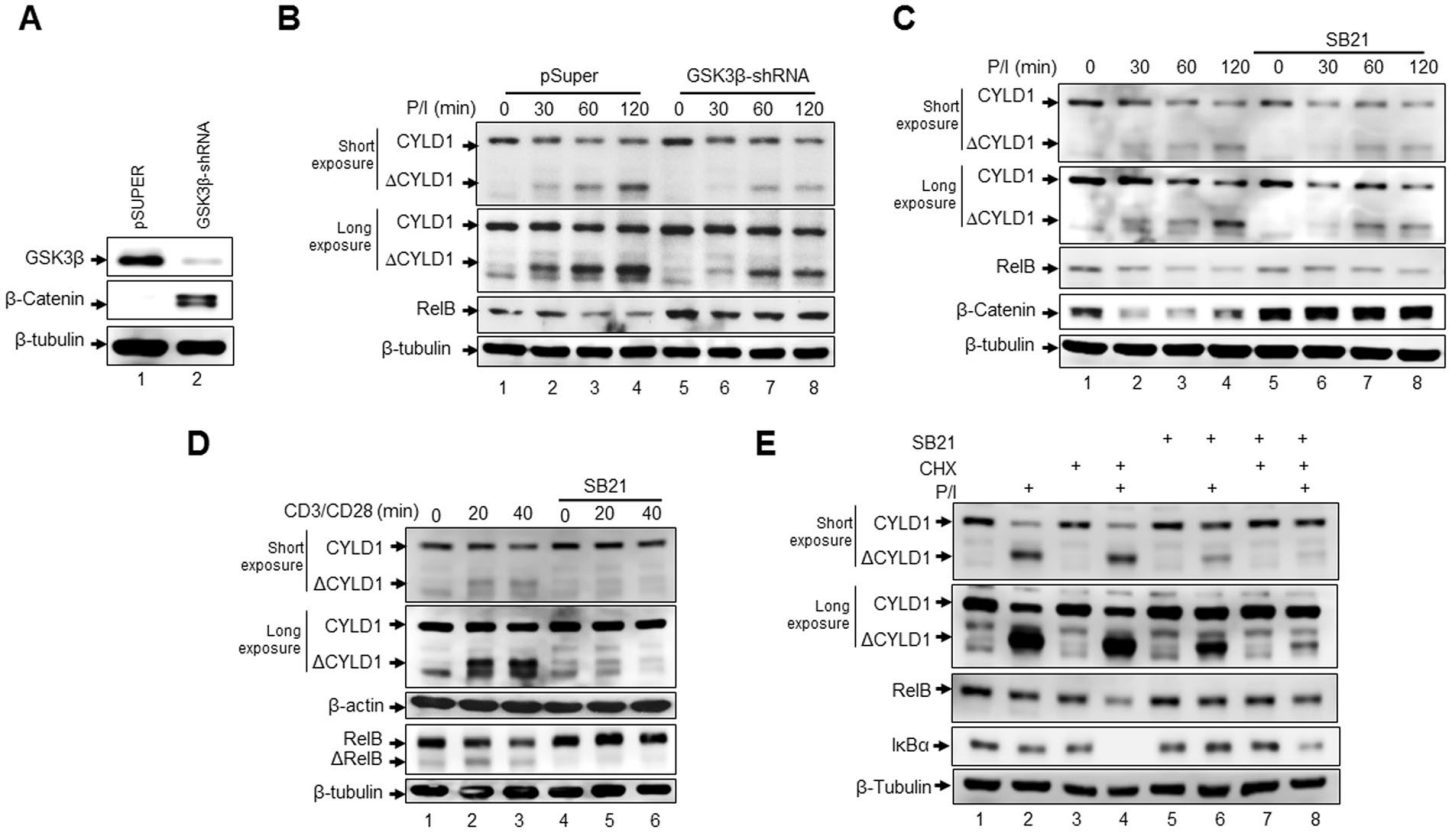
Inhibition of GSK3β attenuates the activity of the MALT1 para-caspase. (**A**) Immunoblot analysis of the indicated proteins in stable Jurkat T-ALL cell clones expressing either a control shRNA (pSUPER) or a GSK3β specific shRNA (GSK3β-shRNA). (**B**) Immunoblot analysis of CYLD1, RelB and β-tubulin in the Jurkat-shControl or the Jurkat-shGSK3β cells. The cells were either left untreated (lanes 1 + 5) or were stimulated with P/I for the indicated times (lanes 2–4 + 6–8). ΔCYLD1 = CYLD1 cleavage product. (**C**) Immunoblot analyses for the indicated proteins using whole cell extracts from Jurkat cells either with or without SB21 pre-treatment prior to a stimulation with P/I for the indicated times. (**D**) Immunoblot analysis of the CYLD1, β-actin, RelB and β-tubulin in Jurkat T-ALL cells stimulated with agonistic anti-CD3/ CD28 antibodies for the indicated times. ΔCYLD1, ΔRelB: cleavage product of CYLD1 and RelB, respectively.

A recently published study demonstrated a crucial role of GSK3β for signal-induced RelB degradation in T cells with RelB forming a protein complex with GSK3β even in unstimulated Jurkat T-ALL cells^15^. Moreover, the initial endoproteolytic step of the RelB degradation, removing the amino terminus of RelB, has been demonstrated to be mediated by the para-caspase MALT1, cleaving RelB after Arg^85 ^^11^. In order to determine whether GSK3β activity has an impact on the proteolysis of other MALT1 substrates, we established Jurkat T-ALL cell clones either stably expressing a control shRNA (Jurkat-shControl cells) or a GSK3β-specific shRNA (Jurkat-shGSK3β cells) leading to a distinct reduction of GSK3β expression and to increased β-catenin protein levels (Fig. 1A, Supplemental Fig. 1A). When stimulated with P/I, RelB levels decreased in Jurkat-shControl cells, as expected (Fig. 1B, lanes 1–4). By contrast, RelB degradation was attenuated in Jurkat shGSK3β cells supporting the previously reported regulatory role of GSK3β for RelB degradation (Fig. 1B, lanes 5–8). Similarly, P/I stimulation of Jurkat-shControl cells caused a distinct proteolysis of CYLD1, which is indicated by the reduction of the full length CYLD1 with a molecular weight of approximately 120 kDa and the appearance of an additional CYLD1 specific signal at ≈70 kDa (ΔCYLD1), representing the C-terminal cleavage product of CYLD1 (Fig. 1B, upper panel). Interestingly, Jurkat-shGSK3β cells showed reduced levels of the CYLD1 cleavage product even after 120 minutes P/I stimulation. To further confirm this effect of GSK3β inhibition on the proteolysis of MALT1 substrates, Jurkat T-ALL cells were stimulated with either P/I (Fig. 1C) or agonistic anti-CD3/CD28 antibodies (Fig. 1D) for different time intervals with and without pre-treatment with the GSK3β inhibitor SB216763 (SB21). Again, GSK3β inhibition caused a decreased formation of ΔCYLD1 and a diminished degradation of RelB. Similar effects on RelB stability was observed upon siRNA-mediated GSK3β suppression (Supplemental Fig. 2B). Moreover, GSK3β inhibition on RelB and CYLD1 degradation were observed in P/I-stimulated HSB2 cells, another T-cell acute lymphoblastic leukaemia (T-ALL) cell line (Supplemental Fig. 1B). The β-catenin levels were stabilized upon SB21 pre-treatment of P/I-stimulated Jurkat cells underscoring the efficacy of the GSK3β inhibition achieved by SB21 pre-treatment. To exclude the possibility that the reduced ΔCYLD1 levels observed upon SB21-mediated GSK3β inhibition are due to a diminished basal CYLD1 expression, we included the translation inhibitor cycloheximide (CHX) in an additional similar experiment. Pre-treatment of Jurkat T-ALL cells with CHX had only a minor effect on the expression levels of full-length CYLD1 (Fig. 1E, compare lanes 1, 3, 5, and 7). Moreover, attenuation of ΔCYLD1 formation by SB21 was also observed in the samples with CHX pre-treatment (Fig. 1E, compare lanes 1 + 2 and 5 + 6 with lanes 3 + 4 and 7 + 8). CHX pre-treatment had also no effect on the stabilization of RelB by SB21-mediated GSK3β inhibition. However, CHX pre-treatment appears to have a general effect on ΔCYLD1 formation, independent of GSK3β activation levels. Taken together, these results imply that GSK3β is required for signal induced MALT1 endoprotease activity in T-ALL cell lines.

### GSK3β modulates the CBM complex formation

**Figure 2:**
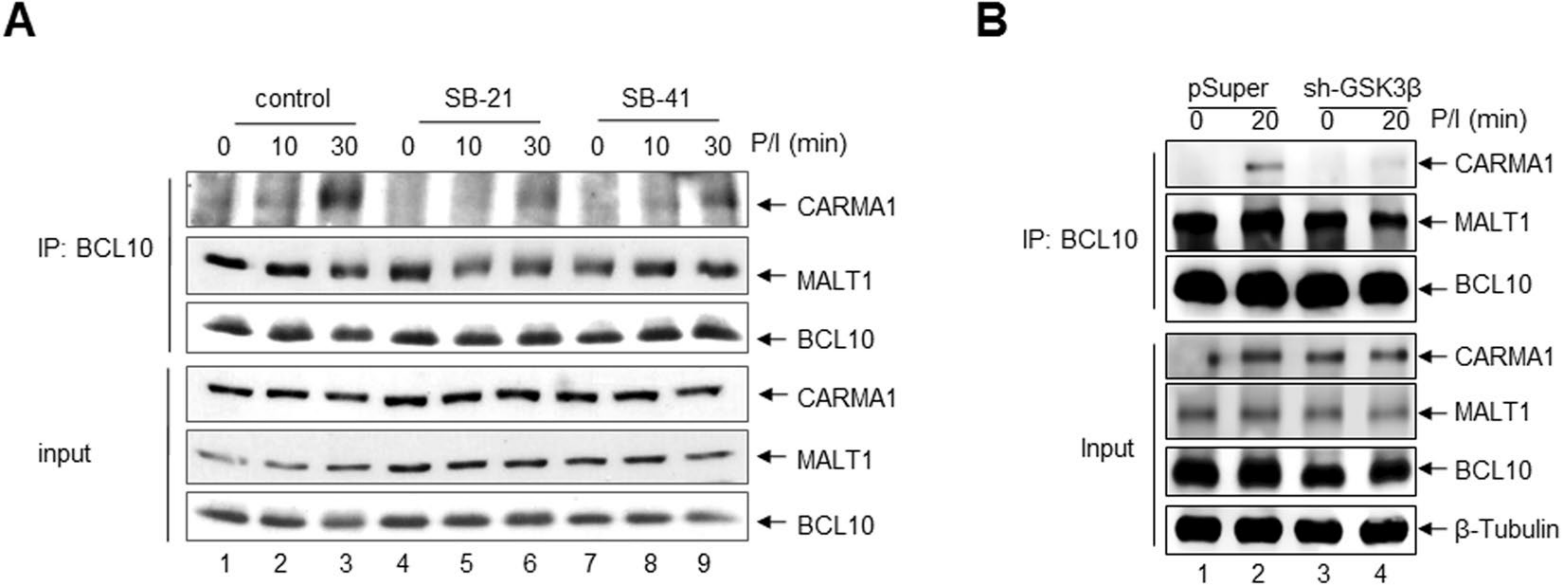
The P/I-induced CBM complex formation is diminished upon GSK3β inhibition. (**A**) Whole cell extracts from untreated or P/I stimulated Jurkat T-ALL cells were subjected to an anti-BCL10 immunoprecipitation analysis (upper part). The resulting protein complexes were subjected to immunoblot analysis. The same whole cell extracts were used for additional immunoblot analyses to control protein expression levels (lower part, input). (**B**) Whole cell extracts from untreated or P/I stimulated Jurkat-shControl or the Jurkat-shGSK3β cells were subjected to an anti-BCL10 immunoprecipitation analysis (upper part). The same whole cell extracts were used for additional immunoblot analyses to control protein expression levels (lower part, input).

As the formation of the CBM complex is a pre-requisite for the activation of MALT1, we next asked whether the formation of the CBM complex is affected by GSK3β inhibition. For this, Jurkat T-ALL cells were either left untreated or were pre-treated with SB21 or SB415286 (SB41) prior to P/I stimulation and the resulting whole cell extracts were subjected to BLC10 immunoprecipitation coupled to anti-CARMA1 immunoblot analyses (Fig. 2A). Without GSK3β inhibition BCL10-CARMA1 interaction was observed after 30 minutes of P/I stimulation (Fig. 2A, lane 3), while pre-treatment with either SB21 or SB41 diminished this interaction (Fig. 2A, lanes 6 + 9). This P/I-induced BCL10-CARMA1 interaction was likewise attenuated in Jurkat-shGSK3β cells as compared to Jurkat-shControl cells (Fig. 2B, lanes 2 + 4). Moreover, a similarly diminished BCL10-CARMA1 interaction was observed in Jurkat T-ALL transiently transfected with a GSK3β-specific siRNA, although to a lesser extend (Supplemental Fig. 2A). Together, these results imply that GSK3β modulates the formation if the CBM-complex in activated T cells.

### GSK3β modulates the canonical NF-κB signalling pathway

**Figure 3:**
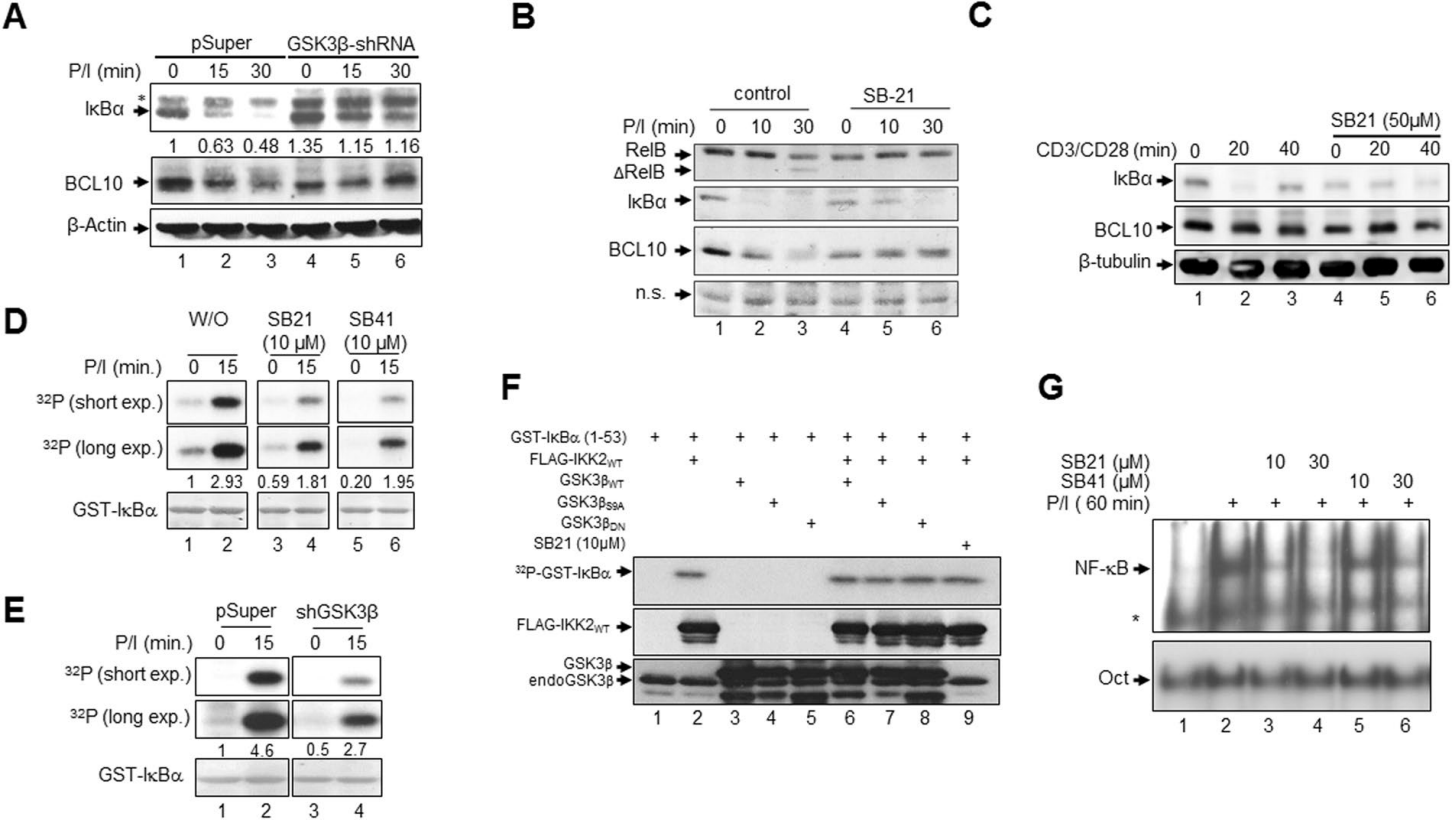
Inhibition of GSK3β affects P/I-induced NF-κB activity. (**A**) Immunoblot analysis of IκBα and BCL10 in P/I-stimulated in the Jurkat-shControl or the Jurkat-shGSK3β cells. (**B**) Immunoblot Analysis of the indicated proteins in Jurkat T-ALL cells with or without pre-treatment with SB21 prior to stimulation with P/I. n.s.: non-specific signal used for control of equal loading of the samples. (**C**) Analysis of IκBα and BCL10 in the same extracts as used in Fig. 1D. (**D**) *In vitro* kinase assay using immunopurified IKK2 from unstimulated or P/I-stimulated Jurkat T-ALL cells without or with SB21 or SB41 pre-treatment. Phosphorylation of the GST-IκBα (1–53) substrate is depicted in the upper and middle panel (^32^P). The equal amount of the recombinant GST-IκBα (1–53) protein is shown by the Ponceau S staining in the lower panel (GST-IκBα). The numbers below indicate the densitometric intensities of the phospho-IκBα signals normalized to the PonceauS signal values. (**E**) A similar *in vitro* kinase assay as described under (**D**) performed with whole cell extracts from Jurkat-shControl or Jurkat-shGSK3β cells. The numbers below indicate the densitometric intensities of the phospho-IκBα signals normalized to the PonceauS signal values. (**F**) *In vitro* kinase assay using ectopically expressed FLAG-IKK2 immunopurified from HEK293 cells transiently transfected as indicated. Cells were either left untreated (lanes 1–8) or were treated with SB21 (lane 9). The phosphorylation of GST-IκBα (1–53) is depicted in the upper panel (^32^P). The expression of ectopic IKK2 and GSK3β is analysed by immunoblot (middle and the lower panel). (**G**) NF-κB DNA-binding study using whole cell extracts from Jurkat T-ALL cells with or without pre-treatment with SB21 or SB41 prior to P/I stimulation in conjunction with a ^32^P-labelled oligonucleotide harbouring a NF-κB consensus site (upper part) or a OCT consensus site (lower part). A band resulting from an unspecific binding is marked by an asterisk.

In order to dissect a potential impact of GSK3β inhibition on the CBM-complex regulated NF-κB activity upon P/I-stimulation, Jurkat-shControl and Jurkat-shGSK3β cells were either left unstimulated or stimulated with P/I for different times, and IκBα and BCL10 protein levels were monitored (Fig. 3A). As expected, P/I stimulation led to marked IκBα degradation in Jurkat-shControl cells, which was distinctively diminished in Jurkat-shGSK3β cells, although these cells displayed higher basal IκBα levels (Fig. 3A, upper panel). Similarly, BCL10 levels, which were decreased in P/I induced Jurkat-shControl cells, were found to be partially stablized in Jurkat-shGSK3β (Fig. 3A, middle panel). Consistently, inhibition of GSK3β by SB21 pre-treatment or by transfection of GSK3β-specific siRNA also hampered IκBα degradation and BCL10 decrease upon P/I (Fig. 3B, Suppl. Fig. 2C) or anti-CD3/CD28 stimulation of Jurkat T-ALL cells (Fig. 3C).

The IκBα stabilization prompted us to ask whether GSK3β inhibition impairs the activity of the IKK complex. Thus, we performed *in-vitro* kinase assay using GST-IκBα (1–53) as a substrate with immunopurified IKK2 from Jurkat T-ALL cells with or without pre-treatment with SB21 or SB41 before stimulation with P/I. In Jurkat cells without GSK3β inhibition, the activity of the IKK complex is drastically increased (Fig. 3D, lanes 1 + 2). Inhibition of GSK3β by pre-treatment with either SB21 or SB41 impaired the activity of the immunopurified IKK complex distinctively (Fig. 3D, lanes 3–6). Similarly, GST-IκBα was found to be less phosphorylated by immunopurified IKK from Jurkat-shGSK3β cells compared to the samples from Jurkat-shControl cells (Fig. 3E). To exclude the possibility that the activity of the major subunit of the IKK complex, IKK2, is affected by GSK3β directly, we performed an *in vitro* kinase assay using extracts from HEK293 cells transiently transfected with an expression vector for FLAG-IKK2 alone or in combination with expression vectors encoding either GSK3β_wt_, GSK3β_S9A_ or GSK3β_K85A_. Additionally, one sample was treated with SB21 to assess the effect of endogenous GSK3β on IKK2 activity. As shown in Fig. 3F, neither the co-expression of the different GSK3β isotypes nor the treatment of the HEK293 cells with SB21 affected the activity of the ectopically expressed FLAG-IKK2. Furthermore, the siRNA-mediated GSK3β-suppression had no impact on the formation of the IKK complex as determined by an anti-NEMO co-immunoprecipitation analysis (Suppl. Fig. 3). The negative impact of GSK3β inhibition, by either pharmacologic inhibitors or siRNA-mediated GSK3β-suppression, on NF-κB DNA-binding activity in P/I-stimulated Jurkat T-ALL cell further underscores the importance of GSK3β for this canonical NF-κB signalling pathway (Fig. 3G, Suppl. Fig. 2D).

### GSK3β inhibition diminishes NF-κB target gene expression

**Figure 4:**
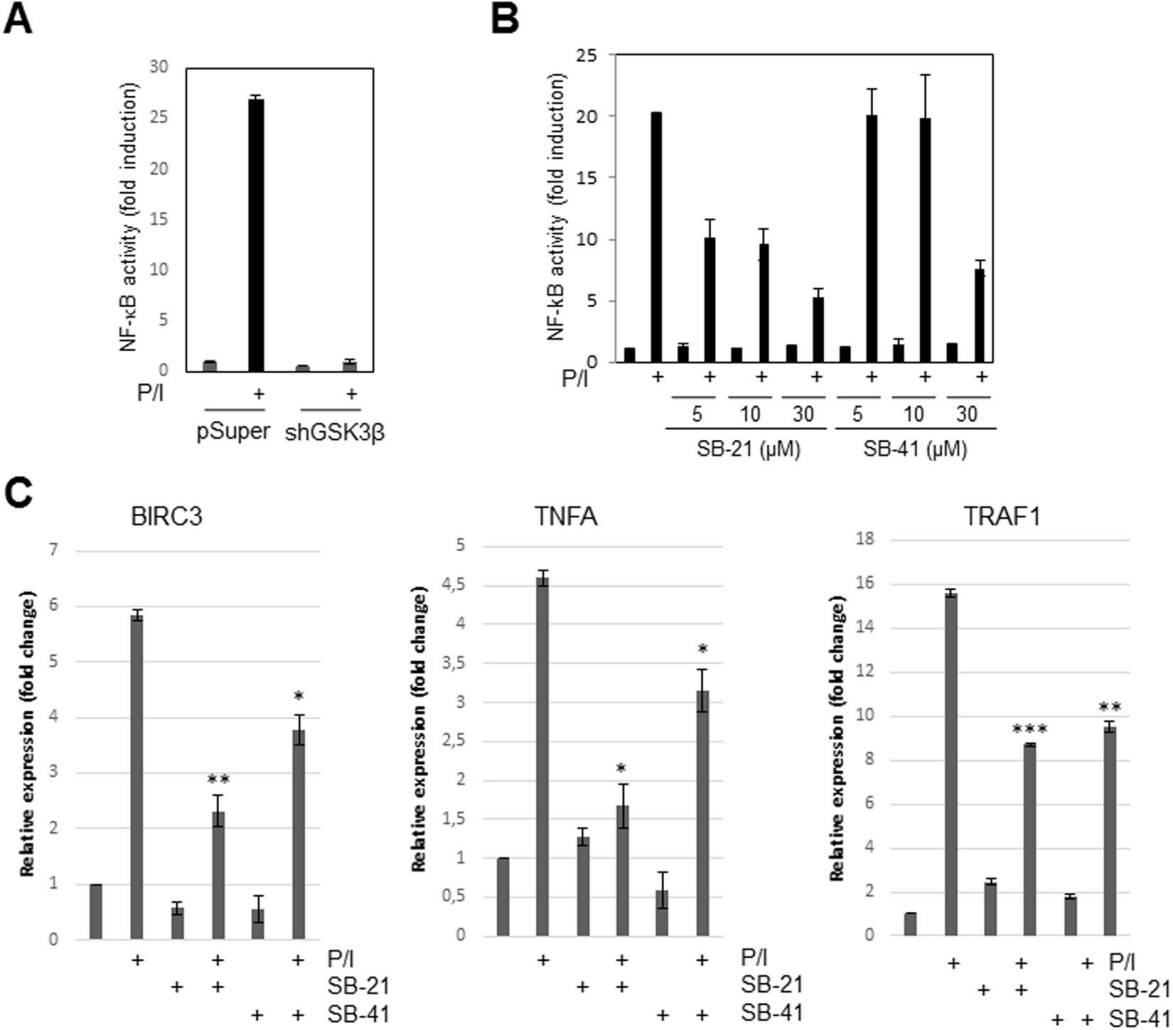
Reduced NF-κB target gene expression upon GSK3β inhibition of Jurkat T-ALL cells. (**A**) Luciferase reporter assay of Jurkat-shControl or the Jurkat-shGSK3β cells transiently transfected with a 3 × κB reporter construct. (**B**) Luciferase reporter assay of Jur4 NF-κB luciferase reporter cells which were either left untreated or were treated with SB21 or SB41 prior to a stimulation with P/I for 6 hours. (**C**) Quantitative real time PCR analyses of mRNA levels of *BIRC3*, *TNFA*, and *TRAF1*. Jurkat T-ALL cells either left untreated or treated with SB21 or SB41 were subjected to a P/I-stimulation for 16 hrs prior to mRNA extraction and analysis. (*p ≤ 0.05, **p ≤ 0.01, ***p ≤ 0.001).

To determine whether the inhibition of GSK3β also affects NF-κB target gene expression, we employed a set of NF-κB luciferase reporter assays using Jurkat-shControl and Jurkat-shGSK3β cells or Jur4 cells. Blocking GSK3β by either GSK3β-specific shRNA (Fig. 4A) or by SB21 or SB41 (Fig. 4B) attenuated the P/I induced NF-κB reporter activity. While P/I stimulation caused a roughly 28 fold increase in NF-κB activity in Jurkat-shControl cells, no significant increase in NF-κB driven luciferase activity was observed in Jurkat-shGSK3β cells (Fig. 4A). Similarly, increasing concentrations of either SB21 or SB41 results caused a dramatic reduction of the P/I induced NF-κB activity in Jur4 cells (Fig. 4B). In addition to the diminished NF-κB-dependent luciferase reporter activity, SB21 or SB41 pre-treatment (Fig. 4C) or the shRNA-mediated knock down of GSK3β (Supplemental Fig. 4) also attenuated the expression of endogenous NF-κB target genes like BIRC3 and TNFA. The expression of TRAF1 was significantly inhibited by SB21 or SB41 pre-treatment, while only a negative tendency was observed in Jurkat-shGSK3β cells. Taken together, these data suggest that GSK3β is involved in the regulation of the P/I-induced canonical NF-κB signalling pathway.

### GSK3β is a BCL10 kinase

**Figure 5:**
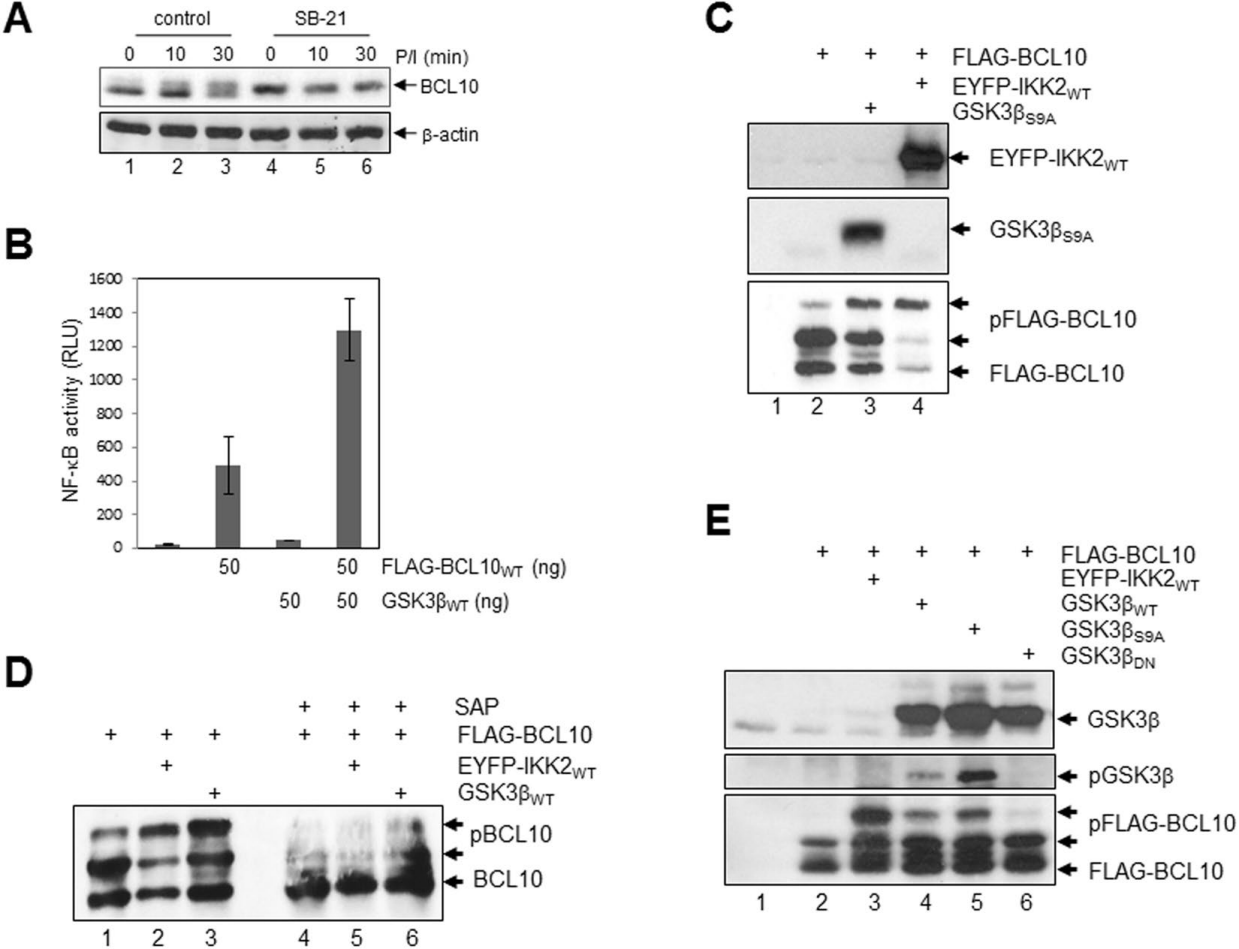
GSK3β regulates the phosphorylation of BCL10. (**A**) Immunoblot analysis of BCl10 levels in P/I-stimulated Jurkat T-ALL cells without (lanes 1–3) or with (lanes 4–6) SB21 pre-treatment. (**B**) NF-κB luciferase reporter assay after transient transfection of HEK293 cells with the indicated plasmids. (**C**) Immunoblot analysis of FLAG-BCL10 ectopically expressed alone or in combination with either IKK2 or GSK3β_S9A_, as indicated. (**D**) Immunoblot analysis of FLAG-BCL10 ectopically expressed alone or in combination with either IKK2 or GSK3β_WT_, as indicated. The immunopurified FLAG-BCL10 proteins were either left untreated (lanes 1–3), or were treated with shrimp alkaline phosphatase (SAP, lanes 4–6) prior to the analysis. (**E**) Immunoblot analysis of FLAG-BCL10 ectopically expressed alone or in combination with either IKK2, GSK3β_WT_, GSK3β_S9A_, or GSK3β_K85A_ as indicated.

To unravel the mechanism underlying the impact of GSK3β on MALT1 activity, CBM complex formation and NF-κB signalling in P/I stimulated Jurkat T-ALL cells, we focused on BCL10 as a potential GSK3β target. IKK2 has been reported to mediate a signal-induced BCL10 phosphorylation upon P/I-stimulation, which is required for the CBM complex formation and NF-κB activation^7^. This BLC10 phosphorylation, marked by the appearance of a P/I induced slower migrating BCL10 isoform, is distinctively reduced in P/I stimulated Jurkat T-ALL cells pre-treated with SB21 (Fig. 5A) suggesting that GSK3β might modulate CBM complex formation by regulating BCL10 phosphorylation. This notion is supported by the synergistic effect of ectopically expressed BCL10 and GSK3β on the NF-κB activity in transiently transfected HEK293 cells, as measured by luciferase reporter assay (Fig. 5B). While BCL10 expression alone had a substantial impact on NF-κB activity, GSK3β expression only mildly induced NF-κB activity. However, addition of GSK3β distinctively augmented the BCL10 caused NF-κB activation. To further characterise the mechanism by which GSK3β augments BCL10-induced NF-κB activation, a FLAG-tagged BCL10 protein was ectopically expressed in HEK293 cells either alone or with IKK2 or GSK3β and BCL10 was monitored by western blot analysis. Besides a fast migrating BCL10 variant several slower migrating BCL10 signals are detectable, potentially representing different phospho-BCL10 isoforms (Fig. 5C, lane 2). With co-expression of IKK2, the slowest migrating BCL10 signal was intensified (Fig. 5C, lane 4), while the faster migrating BCL10 signals were found to be reduced, indicating an IKK2-induced BCL10 proteolysis, as already been reported^7^. With the co-expression of GSK3β similar changes to BCL10 signals were observed, albeit to a lesser extend (Fig. 5C, lane 3). To determine whether the slower migrating BCL10 signals are phospho-BCL10 isoforms, FLAG-BCL10 immuno-purified from HEK293 cells transiently transfected with expression vectors encoding FLAG-BCL10 either alone or in conjunction with IKK2 or GSK3β, were either left untreated or pre-treated with shrimp alkaline phosphatase (SAP) before BCL10 immunoblot. While the BCL10 migration pattern in the control samples was as expected, the slowly migrating bands of BCL10 were strongly reduced in the samples with SAP pre-treatment (Fig. 5D), supporting the idea that these slowly migrating signals representing different phopho-BCL10 isoforms. Moreover, the formation of the slower migrating phospho-BCL10 isoform was further increased when using a constitutive active GSK3β mutant (GSK3β_S9A_), and reduced in case of a dominant negative GSK3β mutant (GSK3β_K85A_), indicating that BCL10 phosphorylation requires the kinase activity of GSK3β (Fig. 5E). Together, these results imply that GSK3β, like IKK2, is a BCL10 kinase.

### IKK2 and GSK3β phosphorylate overlapping serine residues in BCL10

**Figure 6:**
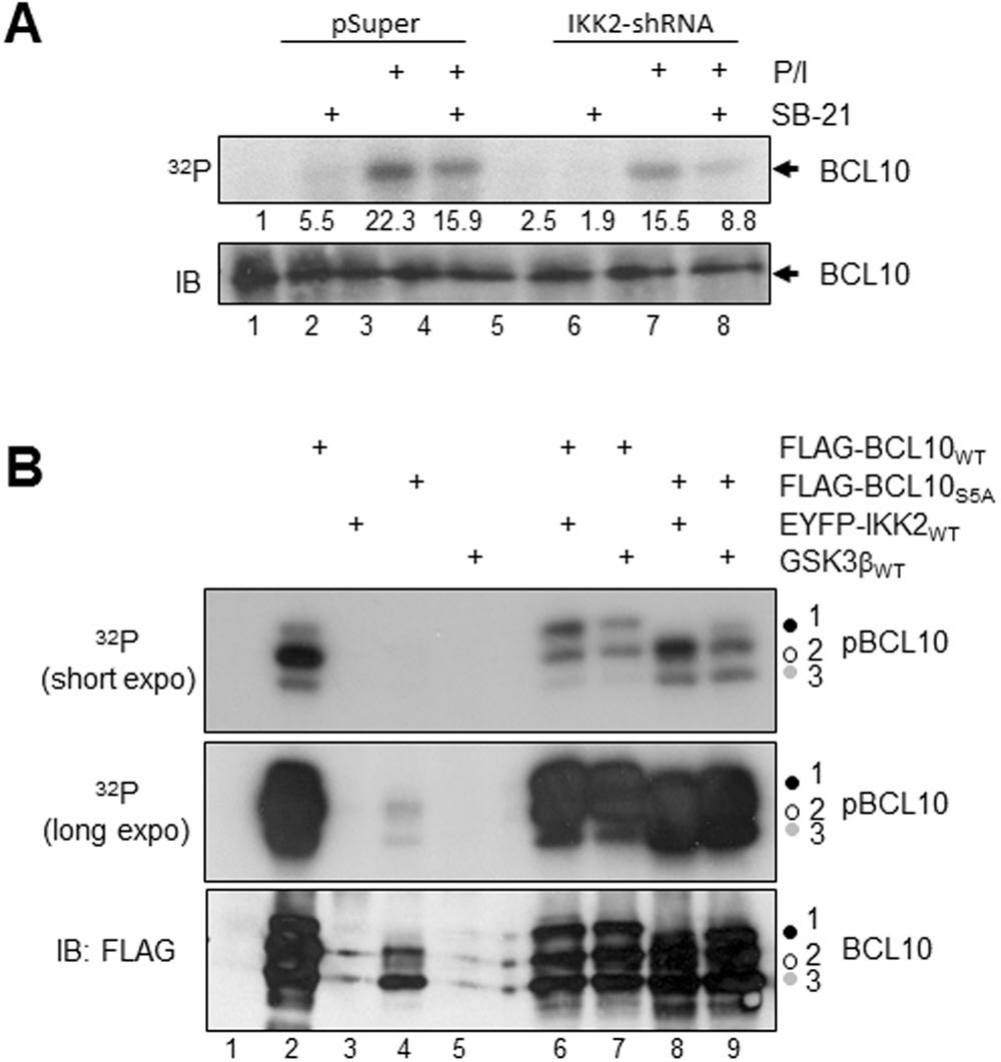
Analysis of IKK2 and GSK3β mediated BCL10 phosphorylation *in vivo*. (**A**) *In vivo* phosphorylation of BCL10 in either Jurkat-shControl cells (pSuper), or in Jurkat-shIKK2 cells (IKK2-shRNA). Cells were metabolic labelled using ^32^P orthophosphate and the BCL10 proteins were subsequently immunopurified and subjected to a SDS-PAGE. (**B**) *In vivo* phosphorylation of FLAG-BCL10_WT_ or FLAG-BCL10_S5A_ ectopically expressed in HEK293 cells either alone or in combination with IKK2 or GSK3β_WT_. Phosphorylation of the immunopurified FLAG-BCL10 is depicted in the upper and middle panels (^32^P). The expression of FLAG-BCL10_WT_ and FLAG-BCL10_S5A_ was determined by anti-FLAG immunoblot staining of the membrane (lower panel).

To further dissect the relationship of GSK3β and IKK2 as BCL10 kinases an *in vivo* phosphorylation experiment was performed using either Jurkat-shControl cells or a Jurkat T-ALL cell line with a stable shRNA-mediated IKK2 knock down (IKK2-shRNA). In accordance to the results shown in Fig. 5, inhibition of GSK3β by SB21 (Fig. 6A, lanes 3 + 4) or the shRNA-mediated knock down of IKK2 (Fig. 6A, lanes 1 + 3 and 5 + 7) diminished the P/I-induced BCL10 phosphorylation in metabolically labelled Jurkat T-ALL cells. Moreover, a pronounced reduction of the P/I-induced BCL10 phosphorylation was observed after a combined inhibition of IKK2 and GSK3β in SB21 pre-treated Jurkat-shIKK2 T-ALL cells (Fig. 6A, lanes 7 + 8). IKK2 targets at least five serine residues in the centre of the BCL10 protein (Ser^134^, Ser^136^, Ser^138^, Ser^141^, and Ser^144^) and a BCL10 mutant with serine-to-alanine substitutions at these positions (BCL10_S5A_) shows an augmented T cell signalling and NF-κB response in reconstituted BCL10-deficient thymocytes^7^. To analyse whether GSK3β phosphorylates the very same serine residues in BCL10, we performed an *in vivo* labelling experiment with HEK293 cells ectopically expressing either FLAG-BCL10_WT_ or FLAG-BCL10_S5A_ (Fig. 6B). BCL10_WT_ displayed a strong basal phosphorylation while BCL10_S5A_ was found to be much less phosphorylated. The co-expression of either IKK2 or GSK3β did not lead to a general increase in BCL10_WT_ phosphorylation, however, it increased the intensity of the slowest migrating, hyper-phosphorylated BCL10_WT_ variant (signal 1). In case of BCL10_S5A_, the co-expression of either IKK2 or GSK3β led a strong increase in the overall BCL10 phosphorylation. However, the phosphorylation patterns caused by IKK2 or GSK3β differed slightly. Together, these results imply that IKK2 and GSK3β can phosphorylate BCL10 at sites additional to the previously reported serine residues.

## Discussion

The formation of a protein complex composed of the CBM complex is a key event in the NF-κB signalling pathway triggered by antigen receptor ligation on lymphocytes. Following the formation of the CBM complex, MALT1 acts as a scaffolding protein which mediates recruitment of downstream effector proteins required for the subsequent activation of the canonical NF-κB signalling^8^. The enzymatic activity of the para-caspase MALT1 proteolytic is required for the endo-proteolytic cleavage of RelB and A20^11,21^. Both proteins have been demonstrated to act as negative regulators of canonical NF-κB signalling and a proteolytic inactivation of these proteins increase the amplitude and duration of NF-κB signalling^9,10,22^. RelB degradation, on the other hand, has been reported to be regulated by the GSK3β-mediated phosphorylation of a serine residue at position 573^15^. RelB:GSK3β protein complexes are formed already in resting Jurkat cells and RelB-bound GSK3β appears to be phosphorylated at Tyr^216^ upon PMA + ionomycin stimulation. The authors of this study showed that the inhibition of GSK3β activity by either pharmacological inhibition or siRNA mediated GSK3β suppression diminishes RelB phosphorylation at Ser^573^ as well as RelB degradation arguing for a direct link of GSK3β-mediated RelB phosphorylation and its proteolysis. On the other hand, an oncogenic CARMA1 mutant from DLBCL cell lines recruits GSK3β to specific high molecular weight protein complex which also includes the CBM complex^16^. Based on these data, we aimed to determine whether GSK3β is capable of modulating CBM complex formation, MALT1 activity and canonical NF-κB signalling in activated lymphocytes. Analysis of the substrates of the MALT1 para-caspase activity in the Jurkat T-ALL model system including CYLD1, BCL10 and RelB revealed a distinct attenuated proteolysis of all tested MALT1 substrates upon GSK3β inhibition either by pharmacological inhibition with SB21 or SB41, or by specific shRNA (Figs 1, 3A–C). In addition, CBM complex formation was attenuated upon GSK3β inhibition either by SB21 or SB41 (Fig. 2A), by specific shRNA (Fig. 2B) or siRNA (Supplemental Fig. 2A). Moreover, GSK3β inhibition also caused a clearly diminished P / I induced NF-κB DNA binding (Fig. 3G and Suppl. Fig. 2D) and NF-κB activity as determined by NF-κB-dependent luciferase reporter assays (Fig. 4A,B) or quantitative RT-PCR of the NF-κB target genes BIRC3, TNFA, and TRAF1 (Fig. 4C, Suppl. Fig. 4).

GSK3β has been shown to affect canonical NF-κB signalling in a variety of cell types. For instance, inhibition of GSK3α and GSK3β blocks the constitutive NF-κB and IKK activity in pancreatic cancer cell lines^23^. By contrast, another study reported that GSK3β affects NF-κB binding to a set of target genes but does not affect IκBα degradation or nuclear NF-κB translocation in pancreatic cancer cell lines^24^. An additional study implies that GSK3β modulates NF-κB activity by directly phosphorylating the NEMO subunit of the IKK complex^25^ or by controlling RelA phosphorylation^20^. However, a direct link of GSK3β to CBM complex formation, MALT activation and NF-κB activation has not been established yet. Although BCL10 has not been reported to bind to the CARMA1:β-catenin protein complex, it is feasible to speculate that BCL10 is recruited to this complex due to its interaction with CARMA1, at least temporary. Alternatively, GSK3β recruitment could also be achieved by its interaction with RelB. As RelB cleavage is mediated by activated MALT1 as part of the CBM complex, a RelB:GSK3β complex bound to MALT1 might be an alternative way to recruit GSK3β to BCL10. As demonstrated by *in vitro* and *in vivo* phosphorylation studies (Figs 5, 6), GSK3β is capable of phosphorylating BCL10 at several serine residues its central part. CBM complex formation is tightly regulated and involves the phosphorylation of all three components of the CBM complex. While CARMA1 is phosphorylated by members of the PKC family, HPK1 and IKK2, BCL10 phosphorylation is exerted by IKK2. Moreover, unlike CARMA1 phosphorylation, which generally promotes CBM complex formation, IKK2-mediated BCL10 phosphorylation appears to be both – a negative as well as a positive regulator of CBM complex formation and NF-κB signalling: While it appears to be an essential step in the initiation of the NF-κB signalling pathway, it is also required for the subsequent shut down of the CBM complex mediated signalling^7^. A process which again involves the MALT1 activity as BCL10 is another MALT1 substrate. The complexity of the functional consequences of BCL10 phosphorylation is further highlighted by the fact that basal BCL10 phosphorylation has to be reduced during the early phase of CBM complex formation by the calcium-dependent phosphatase calcineurin^26^. Therefore, we would like to suggest that GSK3β is another BCL10 kinase similar to IKK2, which is supported by the results of the *in vivo* BCL10 phosphorylation study upon GSK3β and IKK2 inhibition (Fig. 6A). GSK3β and IKK2 might act as redundant systems to ensure BCL10 phosphorylation. Alternatively, GSK3β and IKK2 might phosphorylate a specific, but yet overlapping set of target sites in BCL10 as suggested by the results of the *in vivo* phosphorylation study using exogenous expressed proteins (Fig. 6B). The need for a priming phosphorylation by another kinase is characteristic for GSK3β substrates and has been demonstrated for various substrates including the serum and glucocorticoid-regulated kinase 1 (SGK1) priming phosphorylation of the GSK3β target N-myc downstream regulated gene 1 (NDRG1)^27,28^. Functionally, the GSK3β-mediated BCL10 phosphorylation appears to augment CBM complex formation, NF-κB signalling and MALT1 activation similar to the situation observed with IKK2-mediated BCL10-phosphorylation, suggesting a distinct level of redundancy of both kinases in respect to their role for the antigen-receptor induced NF-κB and potentially JNK activation.

## Methods

### Cell Culture, transfection, and siRNA-mediated knockdown

Jurkat T-ALL cells, HSB2 T-ALL cells, and Jur4 cells were cultivated using an IMEM + RPMI mixture (90:10) supplemented with 10% FCS, glutamine, penicillin, and streptomycin. HEK293 cells were kept in DMEM supplemented with 10% fetal bovine serum, penicillin (50 units/ml), and streptomycin (50 µg/ml). For the stimulation of T cells, 50 ng/ml PMA and 500 ng/ml ionomycin were used. HEK293 cells were transfected using the CaPO_4_ transfection method. In brief, cells were plated the day before transfection to obtain a cell density of about 50% at the day of transfection. For the transfection of one well of a 6-well plate, 1–2.5 µg of DNA was mixed with 90 µl of sterile water and 10 µl of a 2 M CaCl_2_ solution. After incubation for 5 min, 100 µl of 2 × HeBS buffer was added, mixed, and incubated at room temperature for an additional 5 min. Subsequently, the transfection sample was added to the cells. For the suppression of GSK3β by siRNA, Jurkat T cells were transfected using the Nucleofection Kit V (Amaxa/ Lonza). Jurkat T-ALL cells (1 × 10^6^) were transfected with either 1 µl of a 100 nM control siRNA solution, or with 1 µl of a GSK3β-specific siRNA solution (100 nM). The cells were subsequently incubated for 72 h prior to analysis. For the generation of the Jurkat-shControl and Jurkat-shGSK3β cells, Jurkat T-ALL cells were transfected using the appropriate pSUPERpuro constructs and were subsequently selected using 2 µg/ml puromycin.

### Antibodies and reagents

The following antibodies were used in this study: BCL10 (rabbit, sc-5611), BCL10 (goat, sc-9560), IKKα (H-744, sc-7218), IKKα/β (H-470, sc-7607), MALT1 (H-300, sc-28246), NEMO (FL-419, sc-8330), RelB (sc-226), Cylindromatosis-1/CYLD1 (E-10, sc-74435) were from Santa Cruz Biotechnology (Santa Monica, CA, USA). IκBα (44D4, #9242), CARD11/CARMA1 (1D12, #4435), GSK3β (27C10, #9315), GSK3α (#9338) were obtained from Cell Signaling (Danvers, MA, USA). ImmunoCult^TM^ Human CD3/CD28 T cell activator (#10971) was obtained from STEMCELL technologies Inc. (Cologne, Germany). Polyclonal Goat Anti-Mouse Ig (#553998) and Purified Mouse anti-β-catenin (#610154) were obtained from BD Biosciences (San Jose, CA, USA). β-Tubulin (TUB 2.1, #T4026), the GSK3β inhibitors SB216763 (Sigma-Aldrich, #S3442) and SB415286 (Sigma-Aldrich, #S3567), Cycloheximid (Sigma Aldrich, #C7698), and ionomycin (Sigma Aldrich, #I0634) were from Sigma (Sigma-Aldrich, St. Louis, MO, USA). Control siRNA (#SR-CL000-005), GSK3βsiRNA1 (5′-GACUAGAGGGCAGAGUAAAU-3′) and GSK3βsiRNA2 (5′-CCGGGAACAAAUCCGAGAGAU-3′) were obtained from Eurogentec (Liege, Belgium). PMA was purchased (#524400) from Merck (Darmstadt, Germany).

### Plasmids

The pSUPER-GSK3β vector was cloned by inserting either the GSK3β specific oligonucleotide in frame into the HindIII and BglII restriction sites of the pSUPERretro_puro vector. The NF-κB-dependent luciferase reporter construct (3 × κB) and the renilla luciferase reporter construct under the control of the ubiquitin-promoter have been described elsewhere^26^. Expression vectors encoding FLAG-BCL10_WT_ or FLAG-BCL10_S5A_ were reported previously^7^. HA-GSK3β_WT_ pcDNA3 (Addgene plasmid # 14753), HA-GSK3β_S9A_ pcDNA3 (Addgene plasmid # 14754), HA-GSK3β_K85A_ pcDNA3 (Addgene plasmid # 14755) were gifts from Jim Woodgett^29,30^. FLAG-IKK2 and EYFP-IKK2 encoding plasmids are described elsewhere^26^.

### Immunoprecipitation and immunoblotting

Immunoprecipitation and immunoblotting procedures were performed as described previously^26^. In brief, 250–500 µg of protein extracts were mixed with 1 µg/sample of the appropriate antibody, and samples were incubated overnight at 4 °C with agitation. After incubation, 10 µl of a 50% protein G slurry (GE Healthcare) was added, and the samples were further incubated for 1 h. Subsequently, the precipitates were washed extensively in TNT buffer (20 mM Tris, pH 8.0, 200 mM NaCl, 1% Triton X-100, 1 mM DTT, 50 mM NaF, 50 mM β-glycerophosphate, 50 µM leupeptin, 1 mM PMSF). The resulting immunopurified proteins were used for immunoblotting analysis. For the immunoblotting analysis, either the immunopurified protein complexes or, as indicated, 10–50 µg of a protein extract were loaded onto a standard SDS-polyacrylamide gel. SDS-PAGE and the transfer to nitrocellulose (Schleicher & Schuell) were performed using standard protocols. The membrane was blocked with 5% milk powder in TBS + Tween 20 prior to the incubation with the primary antibody (1:1000 in TBS + Tween 20), subsequently washed three times for 5 min each, and incubated in a TBS + Tween 20 solution containing horseradish peroxidase-conjugated secondary antibody (1:5000). The detection was performed using ECL substrates from Pierce/Thermo when exposed to X-ray film or using WesternSure^®^Premium Chemiluminescent Substrate from Li-Cor when using C-Digit blot scanner from Li-Cor. The quantifications were performed using ImageJ 1.49 v or Image studio digits 5.2 software.

### Luciferase Reporter Assay

For the reporter gene assays, a Jurkat T cell clone was used, which was stably transfected with a luciferase reporter gene under the control of a multimerized κB binding site (Jur4 cells,^26^). Cells were treated with the individual reagents as indicated, and luciferase activity was generally estimated after 6 h of treatment. Luciferase values were normalized for protein concentration (relative luciferase units/µg of protein). The experiments were done in duplicates and were repeated at least three times with similar results. For luciferase reporter assay with HEK293 cells, cells were cultivated in 24 well plates and were transiently transfected with 100 ng of the 3 × κB reporter in combination with 15 ng of a renilla luciferase reporter construct under the control of the human ubiquitin promoter. 18 hrs post-transfection, the cells were lyzed and the firefly and renilla luciferase activities were determined according to the protocol of the dual-luciferase system (Promega).

### Gel Shift Analysis

For gel shift analysis (EMSA), 5 µg of nuclear proteins or whole cell extracts (DignamC extracts) from untreated or stimulated cells were incubated on ice for 20 min in a reaction containing 0.3 ng of ^32^P-labeled κB-specific or Oct-specific oligonucleotide, 1 µg of poly(dI:dC), and 3 µl of a 3× binding buffer. The samples were separated on a native 5% polyacrylamide gel, and the gel was dried and subjected to autoradiography.

### *In vitro* kinase assay

For the *in vitro* kinase assays the IKK complex was purified from untreated or P/I stimulated Jurkat T-ALL cells with 1 µg of anti-IKKα/β antibody. Resulting immunocomplexes were washed extensively with TNT and finally with kinase-assay buffer to equilibrate the samples. The kinase reaction was performed at 30 °C for 30 minutes after adding 10 µCi ^32^P γATP and 0.5 µg of a bacterial expressed GST-IκBα (aa1-53) fusion protein in kinase reaction buffer. Samples were subsequently washed extensively with TNT-buffer and PBS prior to a separation by SDS-PAGE. The separated proteins were transferred to nitrocellulose membrane and the phosphorylation was monitored by autoradiography.

### *In vitro* dephosphorylation assay

For the verification of the BCL10 phosphorylation ectopically expressed FLAG-BCL10 was used. After transfection, HEK293 cells were lyzed followed by FLAG-IP with 500 µg/sample. Each sample was split, one was left untreated and the other was treated with 2 units of Shrimp Alkaline Phosphatase (SAP) (Fermentas, #EF0511) in 40 µl total reaction mix. Reaction was performed at 37 °C for 60 min and terminated by incubation at 95 °C for 5 min. Subsequently, the samples were loaded onto a standard SDS-polyacrylamide gel and subjected to immunoblot analysis.

### Real-time PCR analysis

RNA from 2 × 10^6^ cells/sample was isolated using the RNeasy kit (Qiagen) and cDNA was synthesized using M-MLV reverse transcriptase kit (Invitrogen). Real-time PCR analysis to determine BIRC3, TNFA and TRAF1 expression levels was performed using the SYBR green kit form Roche in a Rotor-Gene Q (Qiagen). All measurements were performed in triplicate and the target genes expression were normalized to GAPDH and β-actin expression. The PMA + ionomycin (P/I) induced increase in target genes expression were determined using the ΔΔCt-method.

### Metabolic labelling

For the *in vivo* phosphorylation studies 2 × 10^7^ Jurkat T cells were incubated for 18 hours in phosphate-free DMEM with 5% dialyzed calf-serum prior to incubation with 2 mCi/ml ^32^P orthophosphate for further 6 hours. Resulting whole cell extracts were used for an anti-BCL10 immunoprecipitation analysis as described above. For *in vivo* phosphorylation studies using HEK293 cells, the transiently transfected cells were kept in phosphate-free media including dialyzed FCS for one hour prior to the addition of ^32^P orthophosphate and a further incubation for two hours. The cells were treated as indicated, lyzed in TNT and resulting extracts were subjected to an immunoprecipitation analysis. Precipitated proteins were separated by SDS-PAGE, transferred to nitrocellulose membrane and the resulting membrane was used for an autoradiography to monitor the phosphorylation and subsequently subjected to immunoblot analysis.

### Data availability statement

All data generated or analysed during this study are included in this published article (and its Supplementary Information files).

## Electronic supplementary material

Supplementary Information

## References

[1] Jost P, Peschel C, Ruland J (2006). The Bcl10/Malt1 signaling pathway as a drug target in lymphoma. Current drug targets.

[2] Turvey SE (2014). TheCARD11-BCL10-MALT1 (CBM) signalosome complex: Stepping into the limelight of human primary immunodeficiency. The Journal of allergy and clinical immunology.

[3] Yang C, David L, Qiao Q, Damko E, Wu H (2014). The CBM signalosome: potential therapeutic target for aggressive lymphoma?. Cytokine & growth factor reviews.

[4] Brenner D (2009). Phosphorylation of CARMA1 by HPK1 is critical for NF-kappaB activation in T cells. Proceedings of the National Academy of Sciences of the United States of America.

[5] Matsumoto R (2005). Phosphorylation of CARMA1 plays a critical role in T Cell receptor-mediated NF-kappaB activation. Immunity.

[6] Bidere N (2009). Casein kinase 1alpha governs antigen-receptor-induced NF-kappaB activation and human lymphoma cell survival. Nature.

[7] Wegener E (2006). Essential role for IkappaB kinase beta in remodeling Carma1-Bcl10-Malt1 complexes upon T cell activation. Molecular cell.

[8] Sun L, Deng L, Ea CK, Xia ZP, Chen ZJ (2004). The TRAF6 ubiquitin ligase and TAK1 kinase mediate IKK activation by BCL10 and MALT1 in T lymphocytes. Molecular cell.

[9] Marienfeld R (2001). Signal-specific and phosphorylation-dependent RelB degradation: a potential mechanism of NF-kappaB control. Oncogene.

[10] Coornaert B (2008). T cell antigen receptor stimulation induces MALT1 paracaspase-mediated cleavage of the NF-kappaB inhibitor A20. Nature immunology.

[11] Hailfinger S (2011). Malt1-dependent RelB cleavage promotes canonical NF-kappaB activation in lymphocytes and lymphoma cell lines. Proceedings of the National Academy of Sciences of the United States of America.

[12] Jeltsch KM, Hu D, Brenner S (2014). Cleavage of roquin and regnase-1 by the paracaspase MALT1 releases their cooperatively repressed targets to promote T(H)17. differentiation..

[13] Klein T (2015). The paracaspase MALT1 cleaves HOIL1 reducing linear ubiquitination by LUBAC to dampen lymphocyte NF-kappaB signalling. Nature communications.

[14] Staal J (2011). T-cell receptor-induced JNK activation requires proteolytic inactivation of CYLD by MALT1. The EMBO journal.

[15] Neumann M (2011). Glycogen synthase kinase-3beta is a crucial mediator of signal-induced RelB degradation. Oncogene.

[16] Bognar MK (2016). Oncogenic CARMA1 couples NF-kappaB and beta-catenin signaling in diffuse large B-cell lymphomas. Oncogene.

[17] Hoeflich KP (2000). Requirement for glycogen synthase kinase-3beta in cell survival and NF-kappaB activation. Nature.

[18] Beg AA, Sha WC, Bronson RT, Ghosh S, Baltimore D (1995). Embryonic lethality and liver degeneration in mice lacking the RelA component of NF-kappa B. Nature.

[19] Li ZW (1999). The IKKbeta subunit of IkappaB kinase (IKK) is essential for nuclear factor kappaB activation and prevention of apoptosis. The Journal of experimental medicine.

[20] Steinbrecher KA, Wilson W, Cogswell PC, Baldwin AS (2005). Glycogen synthase kinase 3beta functions to specify gene-specific, NF-kappaB-dependent transcription. Molecular and cellular biology.

[21] Ferch U (2009). Inhibition of MALT1 protease activity is selectively toxic for activated B cell-like diffuse large B cell lymphoma cells. The Journal of experimental medicine.

[22] Marienfeld R (2003). RelB forms transcriptionally inactive complexes with RelA/p65. The Journal of biological chemistry.

[23] Wilson W, Baldwin AS (2008). Maintenance of constitutive IkappaB kinase activity by glycogen synthase kinase-3alpha/beta in pancreatic cancer. Cancer research.

[24] Zhang JS (2014). *Differential activity o*f GSK-3 isoforms regulates NF-kappaB and TRAIL- or TNFalpha induced apoptosis in pancreatic cancer cells. Cell death & disease.

[25] Medunjanin S (2016). GSK-3beta controls NF-kappaB activity via IKKgamma/NEMO. Scientific reports.

[26] Palkowitsch L (2011). The Ca2+-dependent phosphatase calcineurin controls the formation of the Carma1-Bcl10-Malt1 complex during T cell receptor-induced NF-kappaB activation. The Journal of biological chemistry.

[27] Banz VM (2009). Hsp90 transcriptionally and post-translationally regulates the expression of NDRG1 and maintains the stability of its modifying kinase GSK3beta. Biochimica et biophysica acta.

[28] Schmid E (2014). Serum- and glucocorticoid-inducible kinase 1 sensitive NF-kappaB signaling in dendritic cells. Cellular physiology and biochemistry: international journal of experimental cellular physiology, biochemistry, and pharmacology.

[29] He X, Saint-Jeannet JP, Woodgett JR, Varmus HE, Dawid IB (1995). Glycogen synthase kinase-3 and dorsoventral patterning in Xenopus embryos. Nature.

[30] Stambolic V, Woodgett JR (1994). Mitogen inactivation of glycogen synthase kinase-3 beta in intact cells via serine 9 phosphorylation. The Biochemical journal.

