# Supplementary material for: GSK3β modulates NF-κB activation and RelB degradation through site-specific phosphorylation of BCL10
Source: Sci Rep. 2018 Jan 22;8:1352. doi: 10.1038/s41598-018-19822-z (PMC5777991; doi:10.1038/s41598-018-19822-z)

GSK3β modulates NF-κB activation and RelB degradation through site-specific phosphorylation of BCL10

Ali Abd-Ellah[1,3*], Cornelia Voogdt[1*], Daniel Krappmann[2], Peter Möller[1], and Ralf B Marienfeld[1#]

[1]Institute of Pathology, University of Ulm, Albert-Einstein-Allee 23, 89070 Ulm, Germany, [2]Research Unit Cellular Signal Integration, Institute of Molecular Toxicology and Pharmacology, Helmholtz Zentrum München - German Research Center for Environmental Health, Neuherberg, Germany, and [3]Department of Pathology, Qena faculty of medicine, South Valley University, Qena 83523, Egypt.

* These authors contributed equally to this work.

[#]Corresponding author: Ralf B Marienfeld, email: ralf.marienfeld@uni-ulm.de, phone: +49 731 500 56306

Supplemental Figure 1

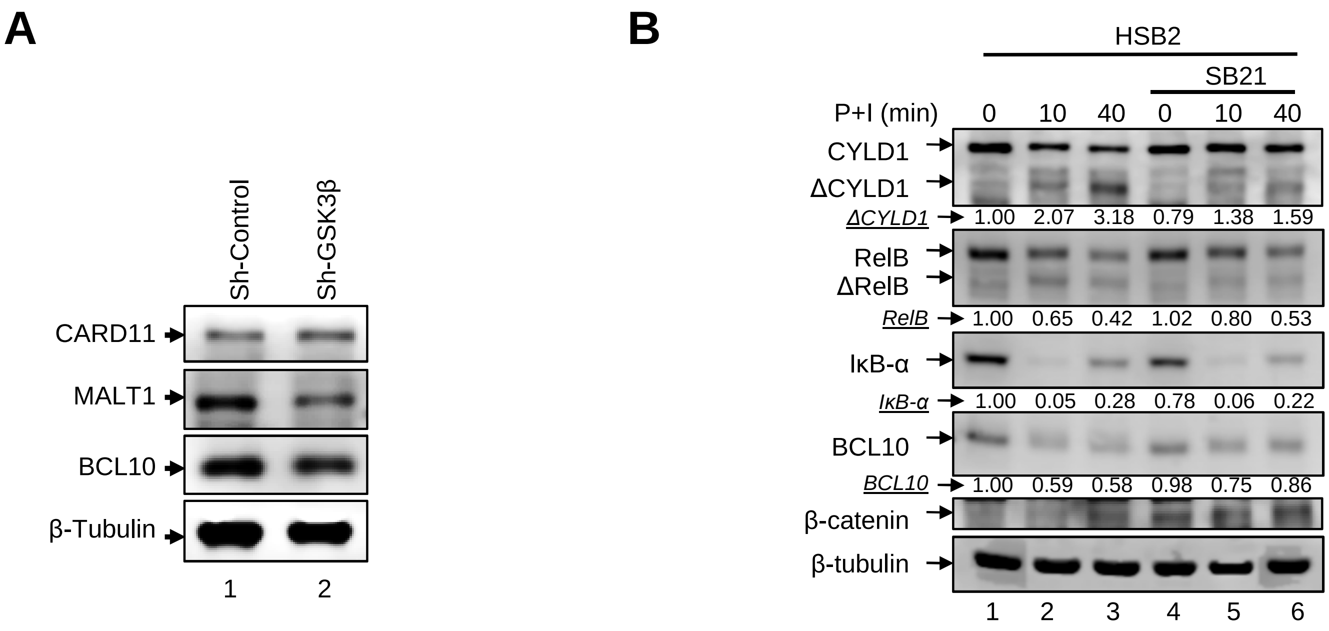

Supplemental Figure 1, *A*, Protein levels of distinct CBM signalling components in control-shRNA or GSK3β-shRNA Jurkat T-ALL cells *B***,** Effects of GSK3β inhibition by SB21 in HSB2 T-ALL cells. Immunoblot analyses for the indicated proteins using whole cell extracts from HSB2 T-ALL cells either with or without SB21 pre-treatment prior to a stimulation with P/I for the indicated times.

Supplemental Figure 2

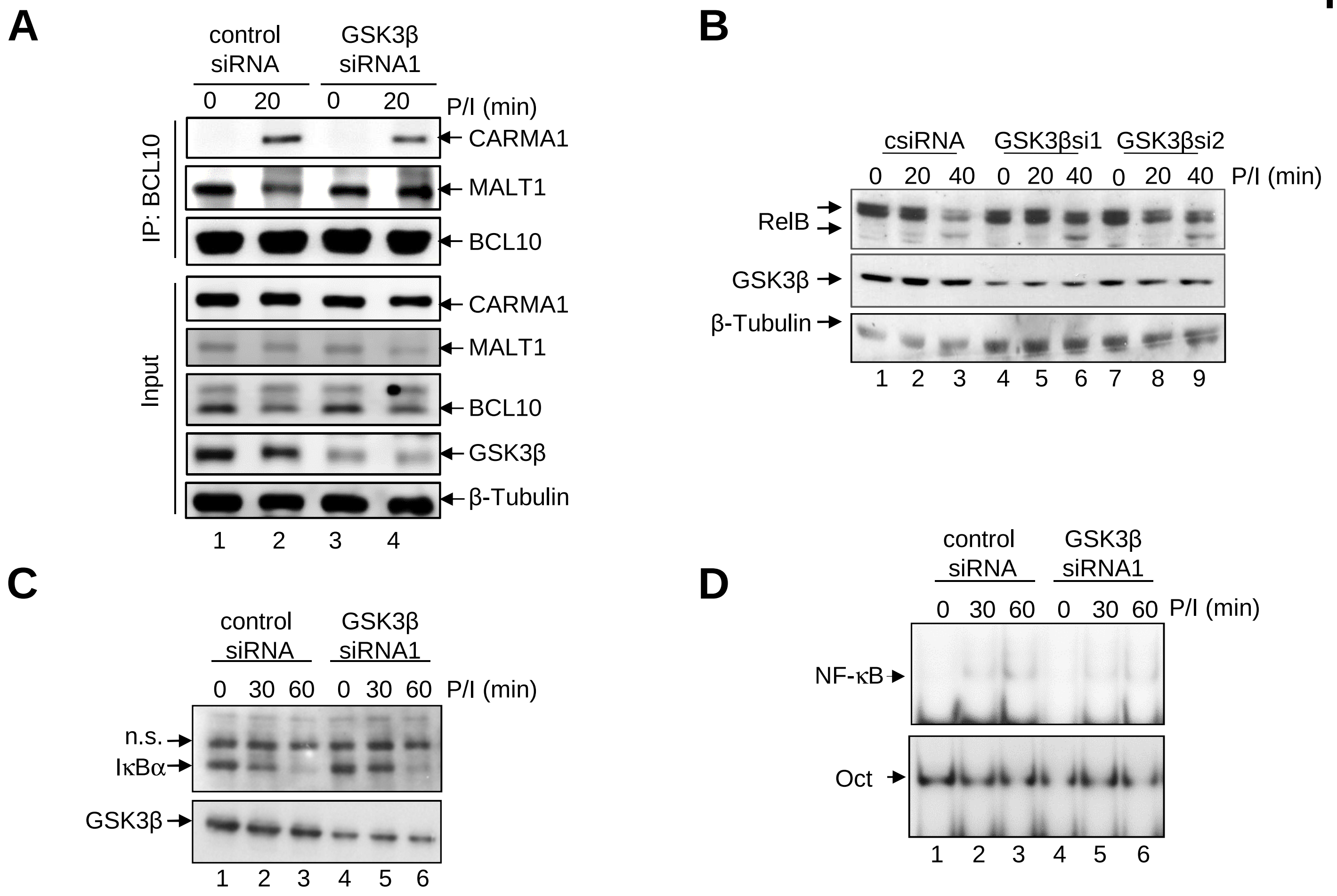

Supplemental Figure 2, Suppression of GSK3β by siRNA attenuates P/I-induced RelB degradation and NF-κB activation. *A,* Jurkat T-ALL cells transiently transfected with either control siRNA (CsiRNA) or with the GSK3β-specific siRNA (GSK3βsiRNA) were subjected to an anti-BCL10 immunoprecipitation analysis (upper part). The same whole cell extracts were used for additional immunoblot analyses to control protein expression levels (lower part, input). *B*, Immunoblot analysis of RelB using whole cell extracts from Jurkat T-ALL cells transiently transfected with either control siRNA, GSK3β siRNA 1 (GSK3βsi1) or GSK3β siRNA 2 (GSK3βsi2) were stimulated with P/I for the indicated times. *C,* Immunoblot analysis of IκBα using Dignam C extracts from Jurkat T-ALL cells transiently transfected with either control siRNA or GSK3βsiRNA 2 (GSK3βsi) were stimulated with P/I for the indicated times. *D*, EMSA experiments with either a κB-specific or an Oct-specific probe were performed using the same samples as described in *C.*

Supplemental Figure 3

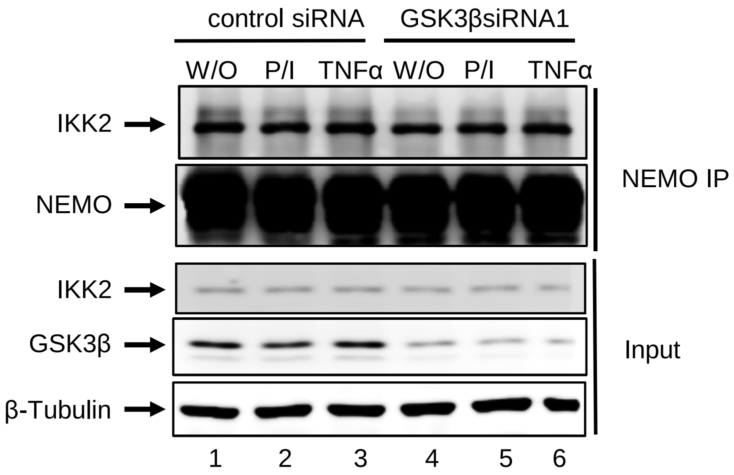

Supplemental Figure 3, IKK complex formation remains unaltered by GSK3β knock-down. IKK complexes were immunopurified form Jurkat T-ALL cells transiently transfected with Control-siRNA (csiRNA) or GSK3βsiRNA 1 (GSK3βsi) using an anti-NEMO antibody. IKK2 and NEMO protein levels were subsequently determined by immunoblot analysis (upper part, NEMO IP). The expression of IKK2 and GSK3β in the input was determined by control immunoblot analyses (lower part).

Supplemental Figure 4

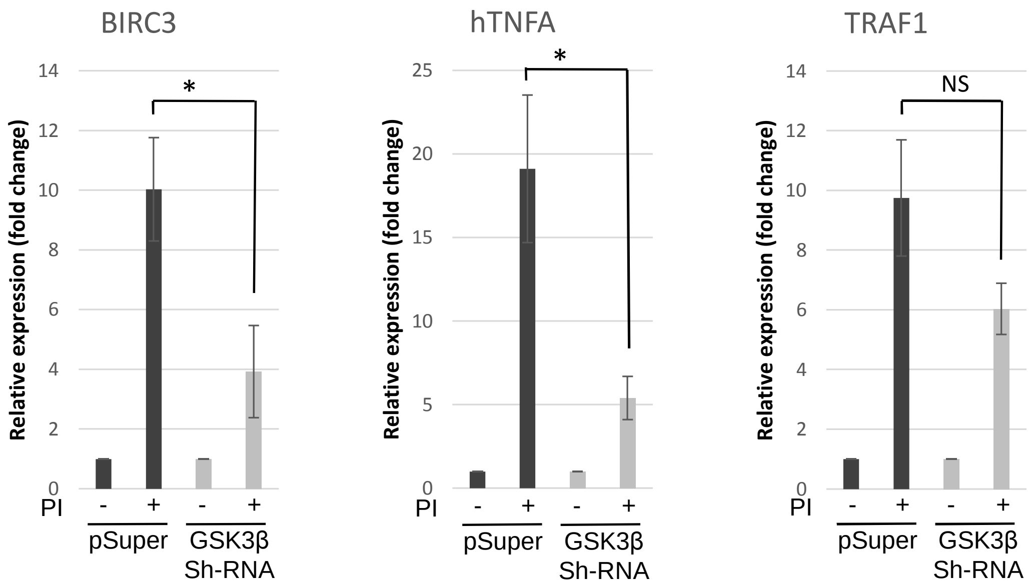

Supplemental Figure 4, Quantitative real time PCR analyses of mRNA levels of BIRC3, TNFA, and TRAF1. The Jurkat-shControl or the Jurkat-shGSK3β cells were subjected to a P/I-stimulation for 16 hours prior to mRNA extraction and analysis. The fold change of unstimulated cells and P/I-stimulated cells is displayed. The Ct values for the control samples were set to 1, arbitrarily. (*p ≤ 0.05, **p ≤ 0.01, ***p ≤ 0.001).

Supplemental Figure 5

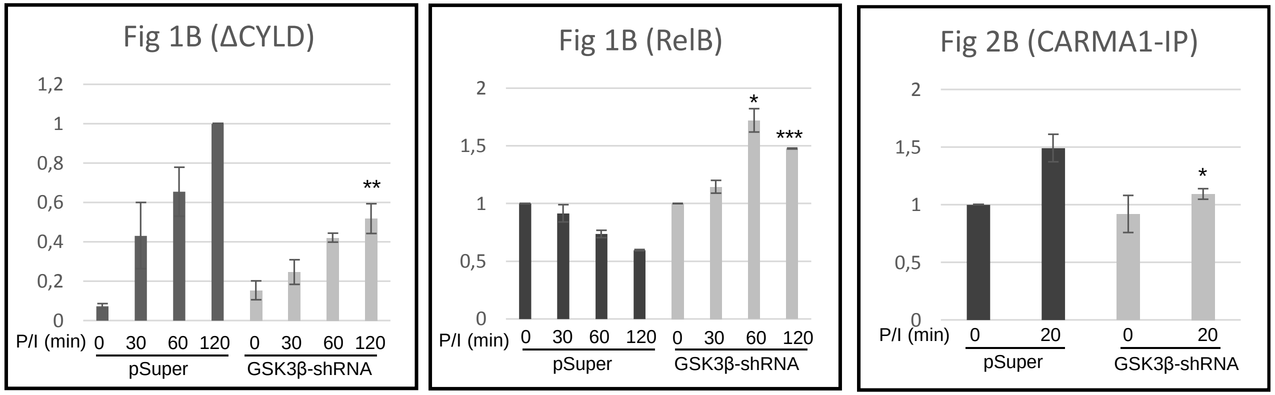

Supplemental Figure 5, Quantifications of selected protein analysis. For the statistical analysis the signals of at least three independent experiments were quantified using either using ImageJ or Image studio digits software. T test analyses were performed using the Graphpad Prism online tool (www.graphpad.com). The title indicates the corresponding figure and the protein quantified (*p ≤ 0.05, **p ≤ 0.01, ***p ≤ 0.001).

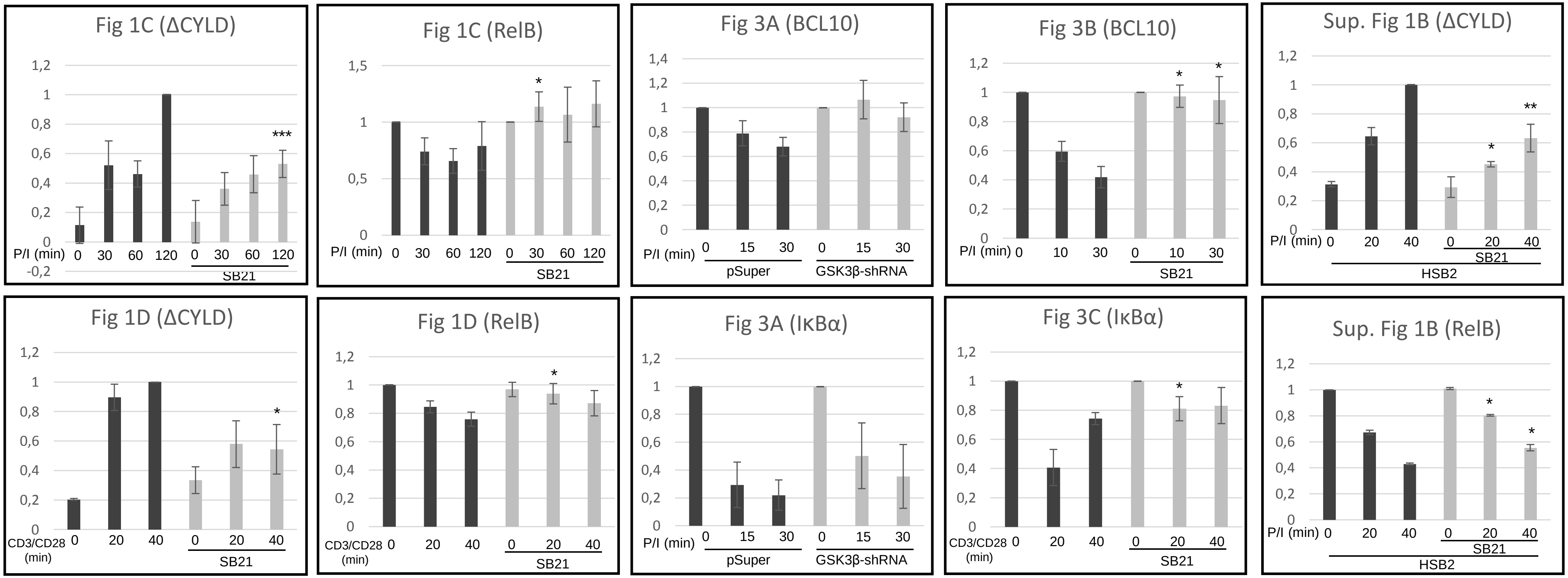

Supplement: Supplementary file 1 — Supplementary Information [file 41598_2018_19822_MOESM1_ESM.pdf]
